# An Original Journal

**Published:** 1880-07

**Authors:** 


AN ORIGINAL JOURNAL.—We are endeavoring to publish, as far as practicable, an *original* journal; will not our kind and generous friends continue to supply us with the communications necessary to accomplish this important object? We trust they will, and thus confer a favor upon our readers as well as upon us, by aiding this desirable consummation.

